# Association of Glucagon-like Peptide-1 Receptor Agonists with Mortality and Aspiration Pneumonia in Patients with Type 2 Diabetes After Gastrostomy: A Target Trial Emulation Study

**DOI:** 10.7150/ijms.128956

**Published:** 2026-03-17

**Authors:** Yuan-Tsung Tseng, Chung-Hung Chen, Li-Ping Chou, Shu-Ying Chen, Huai-Yi Huang, Jyun-Wei Wang, Chung-Yi Li

**Affiliations:** 1 Department of Public Health, College of Medicine, National Cheng Kung University, No. 1, University Road, Tainan City 701, Taiwan.; 2 Department of Medical Research, Tainan Municipal Hospital (Managed by Show Chwan Medical Care Corporation), No. 670, Chongde Road, East District, Tainan City 701, Taiwan.; 3 Division of Gastroenterology, Department of Internal Medicine, Chang Bing Show Chwan Memorial Hospital, No. 6, Lugong Road, Lugang Township, Changhua County 505, Taiwan.; 4 Nursing Department, Nursing College, Hung Kuang University, No. 1018, Section 6, Taiwan Boulevard, Shalu District, Taichung City 433304, Taiwan.; 5 Division of Cardiology, Department of Medicine, Tainan Sin-Lau Hospital, No. 57, Section 1, Dongmen Road, East District, Tainan City 701001, Taiwan.; 6 Department of Health Care Administration, Chang Jung Christian University, No. 1, Changda Road, Gueiren District, Tainan City 711301, Taiwan.; 7 Department of Nursing, Jenteh Junior College of Medicine, Nursing and Management, No. 79-9, Sha-Luen Hu, Xi-Zhou Li, Houlong Township, Miaoli County 356, Taiwan.; 8 Department of Public Health, College of Public Health, China Medical University, Taichung, Taiwan, No. 91, Hsueh-Shih Road, Taichung 40402, Taiwan.; 9 Department of Healthcare Administration, College of Medical and Health Science, Asia University, No. 500, Lioufeng Rd., Wufeng, Taichung 41354, Taiwan.

**Keywords:** glucagon-like peptide-1 receptor agonists, gastrostomy, all-cause mortality, target trial emulation

## Abstract

**Background:**

This study aimed to compare the safety and effectiveness of glucagon-like peptide-1 receptor agonists (GLP-1 RA) versus dipeptidyl peptidase-4 inhibitors (DPP-4i) in patients with type 2 diabetes following gastrostomy.

**Methods:**

We conducted a target trial emulation using real-world data. From January 1, 2015, to December 31, 2024, we identified 728 patients who initiated either GLP-1 RA or DPP-4i after gastrostomy. After 1:1 propensity score matching to balance baseline covariates, 364 patients were included in each group. The primary analysis followed an intention-to-treat principle, and follow-up continued until June 30, 2025. Several sensitivity analyses, including landmark analysis and E-value calculation, were performed to assess the robustness of the findings.

**Results:**

Compared with DPP-4i initiation, GLP-1 RA initiation was associated with lower all-cause mortality (adjusted hazard ratio [aHR], 0.71; 95% confidence interval [CI], 0.54-0.93) and aspiration pneumonia (aHR, 0.64; 95% CI, 0.44-0.93).

**Conclusions:**

Our findings indicate that initiation of GLP-1 RA, compared with initiation of DPP-4i, was associated with lower all-cause mortality and a lower risk of aspiration pneumonia in patients with type 2 diabetes following gastrostomy. Further prospective studies are warranted to confirm these findings in this vulnerable population.

## 1. Introduction

Type 2 diabetes mellitus (T2DM) is a globally prevalent chronic condition characterized by significant morbidity and mortality, primarily from cardiovascular complications 1. Contemporary diabetes management has evolved to incorporate GLP-1 RA and DPP-4i as foundational treatment options, reflecting advances in glucose-lowering pharmacotherapy 2, 3. Large-scale cardiovascular outcome trials (CVOTs) have demonstrated that GLP-1 RA improve glycemic control and reduce both major adverse cardiovascular events (MACE) and all-cause mortality in T2DM populations 4-6. Conversely, DPP-4i have shown neutral cardiovascular effects 7.

Despite robust trial data in general diabetes populations, significant uncertainties remain regarding treatment selection in high-risk subgroups who were not represented in landmark cardiovascular outcome studies. Type 2 diabetes patients requiring gastrostomy tubes represent a medically complex population facing substantial mortality risks. These individuals typically present with numerous concurrent health conditions, including malignancies, neurological disorders, and nutritional deficiencies, that contribute to elevated morbidity beyond traditional cardiovascular complications 8, 9.

Choosing between GLP-1 RA and DPP-4i in post-gastrostomy patients presents a clinical dilemma. While GLP-1 RA offer established benefits, their delayed gastric emptying raises concerns about aspiration pneumonia 10, 11, particularly in this frail population already predisposed to aspiration 12. Given the clinical importance of aspiration complications in patients with severe dysphagia, evidence to guide optimal therapeutic decisions is lacking.

To address this gap, we utilized target trial emulation to compare risks and benefits of GLP-1 RA versus DPP-4i in real-world T2DM patients following gastrostomy. We evaluated all-cause mortality (primary), plus MACE and aspiration pneumonia (secondary outcomes) in this complex population.

## 2. Methods

### 2.1 Study Design and Data Source

This analysis employed a retrospective cohort approach with target trial emulation methodology 13, 14. We utilized the TriNetX global health network, which aggregates de-identified patient records from healthcare institutions across multiple countries. The platform provides access to detailed clinical information encompassing patient demographics, diagnostic codes (ICD-10-CM), procedural data, medication histories, and laboratory findings 15. Data de-identification has been validated by certified experts following HIPAA Privacy Rule requirements (Section §164.514(b)(1)), ensuring patient confidentiality and regulatory compliance. Because the dataset is fully de-identified, this study was granted approval by the Institutional Review Board of Show Chwan Memorial Hospital (IRB No: 1141007) with a waiver of informed consent. 16-18

### 2.2 Study Population and Cohort Construction

**Figure 1:**
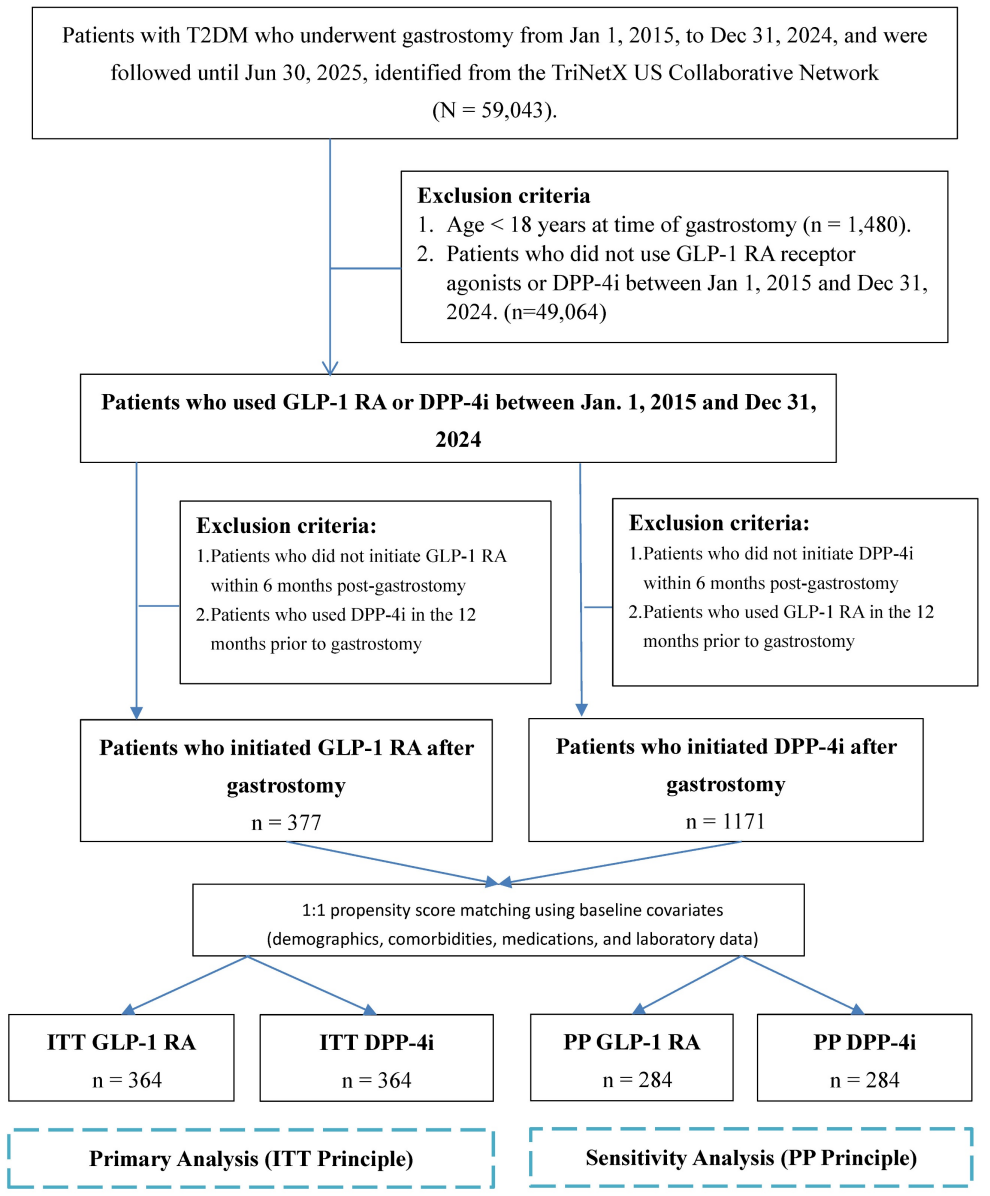
Patient Selection Flowchart for the Intention-to-Treat (ITT) Cohorts. The study cohort was selected from an initial population of 59,043 patients with type 2 diabetes who underwent gastrostomy. An active-comparator design was implemented, defining eligible patients as those who initiated either a GLP-1 RA or a DPP-4i within six months post-procedure. A 12-month washout period was applied, requiring patients to be naive to the comparator drug class in the year prior to gastrostomy. Baseline covariates were captured during a 5-year lookback period before the index date. Subsequently, 1:1 propensity score matching was performed using these covariates (detailed in Table 1), resulting in 364 patients per group. Following the ITT principle, all matched patients were analyzed according to their initial treatment assignment, regardless of subsequent medication changes during follow-up. As a sensitivity analysis, the PP analysis excluded patients who switched between GLP-1 RA and DPP-4i during the follow-up period to evaluate treatment effects among patients who remained on their initial therapy. **Abbreviations**: DPP-4i, dipeptidyl peptidase-4 inhibitors; GLP-1 RA, glucagon-like peptide-1 receptor agonists; ITT, Intention-to-Treat; PP: per-protocol.

Our study cohort comprised 59,043 individuals with type 2 diabetes who received gastrostomy tubes during the period from January 2015 through December 2024 (**Figure 1**). We excluded patients <18 years (n=1,480) and those without GLP-1 RA or DPP-4i use (n=49,064), yielding 8,499 eligible patients.

Following an active comparator design for the overall cohort, we identified patients who initiated either GLP-1 RA or DPP-4i within 6 months after gastrostomy, regardless of whether they had prior use of the index drug before the procedure (i.e., the primary analysis included both new initiators and prevalent users who continued therapy post-gastrostomy). To minimize carryover confounding from the comparator drug class, a 12-month washout period was applied, requiring patients to have no prescription records of the comparator drug in the 12 months preceding gastrostomy. Specifically, the GLP-1 RA group initiated treatment within 6 months post-gastrostomy without DPP-4i use in the 12 months prior to gastrostomy. The DPP-4i group initiated treatment within 6 months post-gastrostomy without GLP-1 RA use in the 12 months prior to gastrostomy.

We performed 1:1 propensity score matching to balance baseline differences, creating final intention-to-treat (ITT) cohorts of 364 patients each. The index date was the first GLP-1 RA or DPP-4i prescription post-gastrostomy 19, 20.

### 2.3 Outcome Measures

Patients were followed from the index date until the occurrence of all-cause mortality, loss to follow-up, or the end of the study observation period. The primary outcome was all-cause mortality. Secondary outcomes included MACE and the incidence of pneumonia. MACE was defined as a composite endpoint of acute myocardial infarction, stroke (ischemic or hemorrhagic), and cardiac arrest. Aspiration pneumonia was identified using diagnosis codes; the database does not allow reliable adjudication of whether events were peri-procedural/anesthesia-related versus feeding-related. Follow-up ended at the first outcome event, death, the last recorded encounter (loss to follow-up), or the end of data availability; apart from recorded death, reasons for study termination cannot be fully distinguished.

### 2.4 Covariates

Baseline patient characteristics were captured during a 5-year lookback period prior to the index date. Covariates included for propensity score matching (PSM) were demographics (age, sex, race), anthropometrics (body mass index, BMI), social and behavioral factors (nicotine dependence, alcohol abuse), major comorbidities (e.g., hypertension, hyperlipidemia, chronic kidney disease, heart failure), concomitant medications (e.g., insulin, metformin, statins, ACE inhibitors/angiotensin II receptor blockers, ARBs), and baseline laboratory data (e.g., hemoglobin A1c; HbA1c, estimated glomerular filtration rate; eGFR, albumin, C-reactive protein). For the purpose of subgroup analysis, a composite variable for atherosclerotic cardiovascular disease (ASCVD) was created, defined as a baseline diagnosis of either ischemic heart disease or cerebrovascular disease (see **Supplementary Table 1** for detailed definitions and codes).

### 2.5 Statistical Analysis

Initial cohorts included 377 GLP-1 RA and 1,171 DPP-4i users. 1:1 PSM used nearest neighbor matching with 0.2 SD caliper to balance covariates, yielding 364 patients per group 21. Primary analysis followed ITT principles. We used Kaplan-Meier curves with log-rank tests and Cox proportional hazards models for adjusted hazard ratios (aHRs) with 95% confidence intervals (CIs).

To assess how the hazard ratios changed over time, landmark analyses were conducted at 30, 60, and 180 days post-index date; for each landmark time point, the analysis included only those patients who were still alive and had not experienced the outcome of interest 22. To evaluate the robustness of our findings against potential unmeasured confounding, an E-value was calculated for the primary outcome 23. A two-sided p-value < 0.05 was considered statistically significant. Primary analyses were performed using the integrated analytical tools of the TriNetX platform. All data visualization and subsequent advanced analyses were conducted using R software (version 4.3.2; R Foundation for Statistical Computing) through the RStudio interface (version 2023.12.0; Posit PBC).

### 2.6 Subgroup and Sensitivity Analyses

To assess the consistency of the associations with the primary outcome, we conducted several prespecified subgroup analyses stratified by the following baseline characteristics: GLP-1 RA user type (long-term vs. new user; defined by whether patients had any prescription record of the index drug at any time prior to gastrostomy), history of ASCVD, history of heart failure, baseline eGFR (< 60 vs. ≥ 60 mL/min/1.73m²), age (< 65 vs. ≥ 65 years), BMI (< 30 vs. ≥ 30 kg/m²), and history of cancer. The potential for effect modification was assessed by including an interaction term in the Cox model.

Furthermore, a series of sensitivity analyses were performed to evaluate the robustness of our findings. First, to account for all-cause mortality as a competing risk for non-fatal outcomes, we analyzed composite outcomes of (1) MACE or all-cause mortality and (2) pneumonia or all-cause mortality using a Cox model 24, 25. Second, to evaluate the stability of our matching strategy, we constructed five different propensity score models with varying levels of covariate adjustment and compared the resulting aHRs. Third, we conducted a per-protocol (PP) analysis that excluded patients who switched from their initial treatment group to the comparator's drug class during follow-up.

### 2.7 Validation Analyses

To evaluate potential residual confounding and selection bias, we conducted several validation analyses. For positive outcome controls, we assessed the risk of biliary-related diseases and gastrointestinal adverse events 26. For negative outcome controls, we assessed the risk of appendicitis, urinary tract infection, and acute pancreatitis 27. Furthermore, for validation, we conducted a positive exposure control comparing statin users to non-users, and a negative exposure control comparing vitamin C users to non-users 28.

## 3. Results

### 3.1 Study Population Characteristics

Our study initially identified 59,043 patients with type 2 diabetes who underwent a gastrostomy procedure from the TriNetX network. After applying exclusion criteria, we established a pre-matched cohort of 377 GLP-1 RA users and 1,171 DPP-4i users. Before matching, significant baseline differences were observed between the groups; for instance, the GLP-1 RA group was younger (mean age 62.9 vs. 67.2 years) and had a higher BMI (30.5 vs. 27.3 kg/m²) compared to the DPP-4i group. Following a 1:1 PSM procedure, two analytical cohorts of 364 patients each were created. PSM was performed using the nearest neighbor approach with a 0.2 SD caliper to achieve the best possible covariate balance (the patient selection process is detailed in Figure 1, and baseline characteristics are compared in **Table 1**).

### 3.1 Primary and Secondary Outcomes

**Figure 2:**
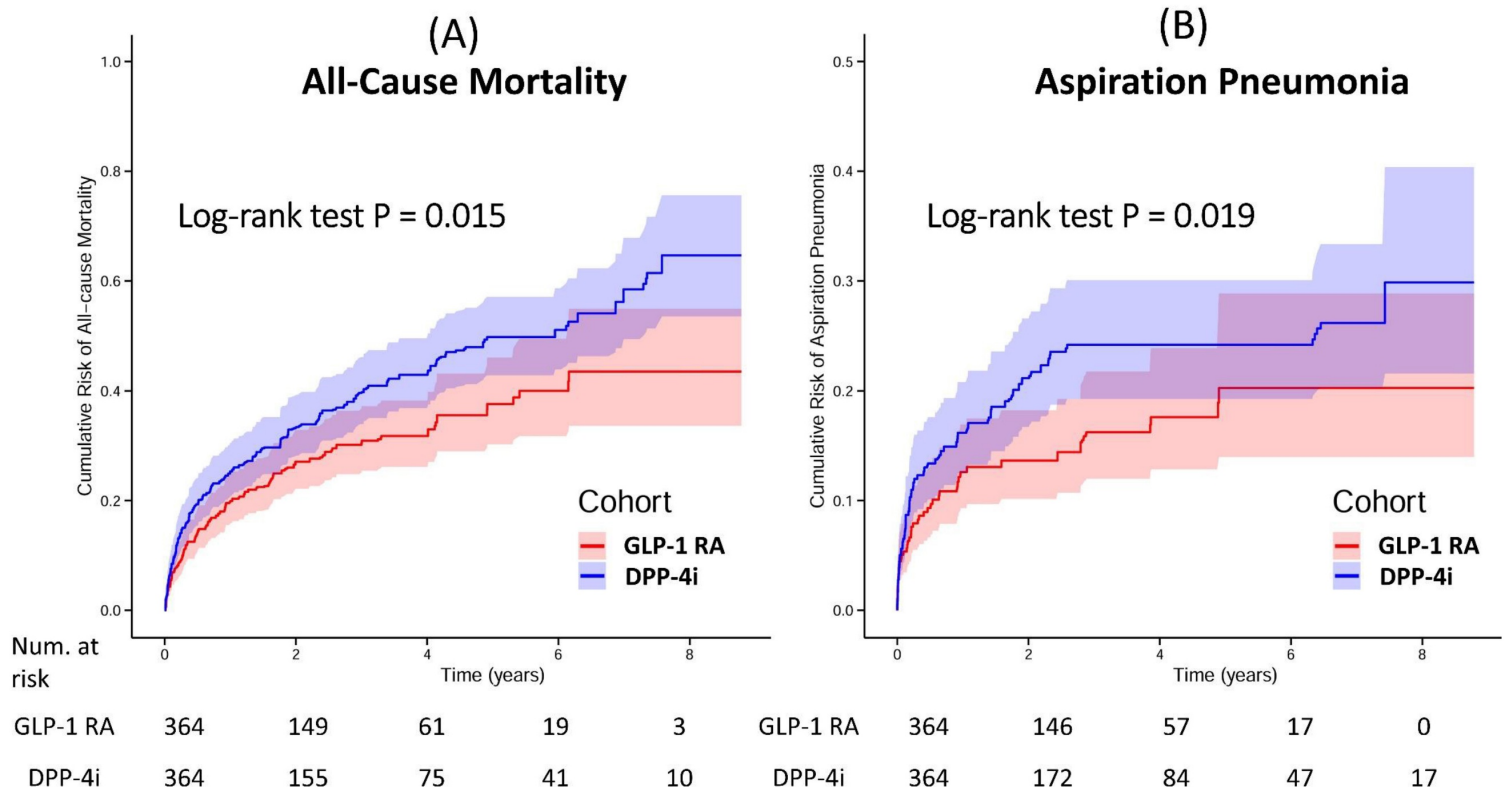
Kaplan-Meier Curves for the Cumulative Risk of All-Cause Mortality and Aspiration Pneumonia in Patients Using GLP-1 RA Versus DPP-4i. Kaplan-Meier curves illustrate the cumulative risk of (A) All-Cause Mortality and (B) Aspiration Pneumonia in propensity score-matched cohorts of patients treated with GLP-1 RA (red line) and DPP-4i (blue line). P-values were derived from the log-rank test to compare the curves. The shaded areas represent the 95% confidence intervals. The number of patients at risk at various time points is provided below each panel. **Abbreviations:** DPP-4i, dipeptidyl peptidase-4 inhibitors; GLP-1 RA, glucagon-like peptide-1 receptor agonists.

Median follow-up was 1.93 years (GLP-1 RA) and 1.73 years (DPP-4i). Kaplan-Meier analysis showed a lower mortality risk with GLP-1 RA (log-rank P = 0.015) (**Figure 2A**).

GLP-1 RA use was associated with lower aspiration pneumonia incidence (aHR: 0.64, 95% CI: 0.44-0.93; log-rank P = 0.019) (**Figure 2B**). No significant difference emerged for MACE (aHR: 1.01, 95% CI: 0.79-1.28).

Overall, the use of GLP-1 RA was associated with a lower risk of the primary outcome, all-cause mortality, compared to the use of DPP-4i (aHR: 0.71, 95% Confidence Interval [CI]: 0.54-0.93).

Landmark analyses revealed temporal variation in the association with mortality, with the strongest association observed after 30 days of follow-up (aHR: 0.63, 95% CI: 0.44-0.89), while associations at 60 and 180 days were not statistically significant. The E-value for the all-cause mortality point estimate was 2.17 (confidence interval limit: 3.11), suggesting robustness against potential unmeasured confounding (Table 2).

### 3.2 Subgroup Analyses

**Figure 3:**
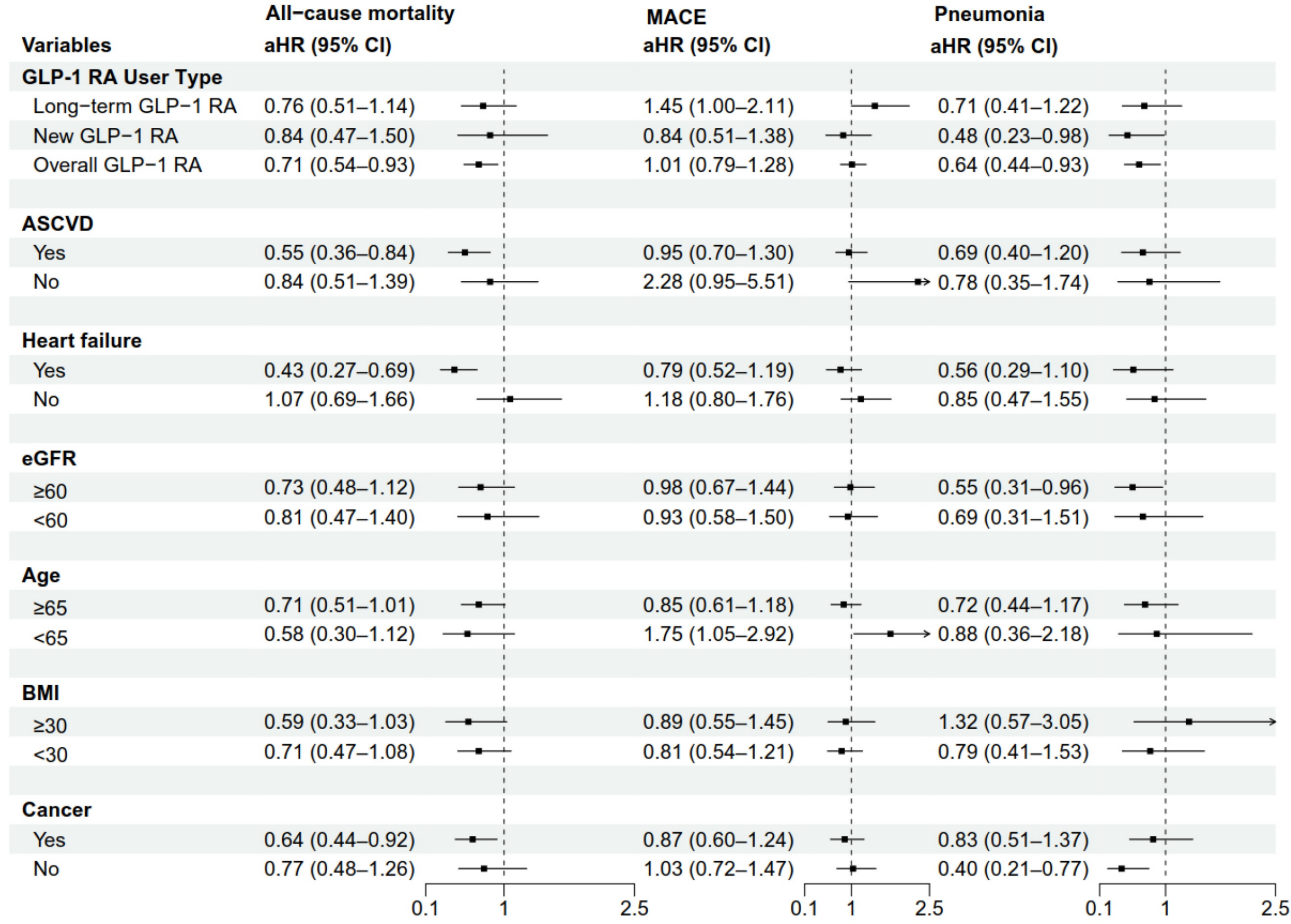
Subgroup Analyses of Mortality, MACE, and Pneumonia for GLP-1 RA Versus DPP-4i. The forest plot displays the adjusted hazard ratios (aHRs) for the use of GLP-1 RA compared with DPP-4i (reference group) on the outcomes of all-cause mortality, MACE, and pneumonia across various prespecified subgroups. The squares represent the point estimates of the aHRs, and the horizontal lines represent the 95% confidence intervals (CIs). A 'New GLP-1 RA user' was defined as a patient with no prior prescription record for GLP-1 RA at any time before gastrostomy, whereas a 'Long-term GLP-1 RA user' was defined as a patient who had an existing GLP-1 RA prescription before gastrostomy and continued use within 6 months post-procedure. **Abbreviations**: aHR, adjusted hazard ratio; ASCVD, atherosclerotic cardiovascular disease; BMI, body mass index; CI, confidence interval; DPP-4i, dipeptidyl peptidase-4 inhibitors; eGFR, estimated glomerular filtration rate; GLP-1 RA, glucagon-like peptide-1 receptor agonists; MACE, major adverse cardiovascular events.

To assess the consistency of the associations, we conducted prespecified subgroup analyses for all-cause mortality, MACE, and pneumonia (**Figure 3**). For all-cause mortality, the association with lower mortality for GLP-1 RA was consistent across multiple subgroups, with a more pronounced trend observed in patients with a history of heart failure (aHR: 0.43, 95% CI: 0.27-0.69), a BMI ≥ 30 kg/m² (aHR: 0.59, 95% CI: 0.33-1.03), and a history of cancer (aHR: 0.64, 95% CI: 0.44-0.92). However, the results for MACE and pneumonia risk varied considerably across subgroups, with most not reaching statistical significance. Notably, among patients younger than 65 years, GLP-1 RA use was associated with an increased risk of MACE (aHR: 1.75, 95% CI: 1.05-2.92). This finding should be interpreted with caution, as it may be a chance occurrence due to the multiple comparisons performed.

### 3.3 Sensitivity and Validation Analyses

To verify the robustness of our findings, we performed a series of sensitivity and validation analyses. For positive outcome controls, we assessed the risk of biliary-related diseases and gastrointestinal adverse events. The results showed no statistically significant difference (aHR 0.96 and 0.81, respectively). We interpret the absence of the expected signal in these positive outcome controls cautiously, as symptom-driven adverse events may be incompletely ascertained in routinely collected EHR data; mild-to-moderate gastrointestinal symptoms are often managed conservatively and may not be consistently captured as diagnosis-coded events unless they lead to distinct medical encounters or interventions, which can reduce sensitivity to detect an expected association. In addition, the sample size (n=364 per group) may be insufficient to detect modest differences in these adverse event rates. In the analysis of negative outcome controls (e.g., appendicitis, urinary tract infection), there was no significant difference in risk between the groups, suggesting the absence of major systemic bias. Crucially, however, the positive exposure control analysis successfully replicated the known mortality benefit of statins versus non-users (aHR: 0.75, 95% CI: 0.69-0.82), while the negative exposure control (Vitamin C) showed no association with mortality (**Supplementary Figure 1**). This suggests that our propensity score matching strategy and data source are valid and sufficiently sensitive to detect true differences in the primary outcome (mortality), despite limited sensitivity for secondary, symptom-driven adverse event signals.

Sensitivity analyses using five different propensity score models with varying levels of covariate adjustment showed that the aHRs for all-cause mortality and aspiration pneumonia remained relatively stable (all-cause mortality range: 0.63-0.76; aspiration pneumonia range: 0.57-0.69)** (Supplementary Table 2)**, supporting the stability of our matching strategy. Finally, the results of the per-protocol analysis were directionally consistent with the primary ITT analysis and suggested a stronger trend of risk reduction for all-cause mortality and aspiration pneumonia (aHR for all-cause mortality: 0.64 vs. 0.71; aHR for aspiration pneumonia: 0.52 vs. 0.64) **(Supplementary Table 3)**, which support the reliability of the study conclusions.

## 4. Discussion

In a unique and vulnerable population of patients with T2DM post-gastrostomy, this study employed a target trial emulation framework to compare the clinical associations and risks of GLP-1 RA versus DPP-4i. The findings delineate a nuanced association profile, providing real-world evidence for this population frequently excluded from large-scale clinical trials.

### 4.1 Summary of Main Findings

Three key findings emerged: First, after PSM, GLP-1 RA was associated with 29% lower all-cause mortality versus DPP-4i (aHR: 0.71, 95% CI: 0.54-0.93). Second, GLP-1 RA was associated with a lower aspiration pneumonia risk (aHR: 0.64, 95% CI: 0.44-0.93), contradicting expectations from delayed gastric emptying. Third, no MACE difference was observed (aHR: 1.01, 95% CI: 0.79-1.28), unlike broader T2DM populations. A subgroup analysis identified a potential signal for increased MACE risk in patients < 65 years (aHR: 1.75, 95% CI: 1.05-2.92); however, as discussed in the Limitations section, this finding requires prospective validation and should not be interpreted as established evidence of differential safety.

### 4.2 Mortality Association in a High-Risk Gastrostomy Cohort

This study demonstrates an association between GLP-1 RA and reduced mortality risk compared to DPP-4i. The direction of this association is consistent with meta-analyses of multiple cardiovascular outcome trials (CVOTs), which have uniformly shown that GLP-1 RA reduce all-cause mortality relative to placebo and are superior to DPP-4i in head-to-head comparisons 29, 30. For instance, a recent meta-analysis reported a pooled HR of 0.82 for all-cause mortality with GLP-1 RA, while another indicated a 12% risk reduction 31.

The association with lower all-cause mortality was consistent across most subgroups, including older adults (Age ≥ 65). Unlike CVOT participants, our post-gastrostomy cohort faces high risks of non-cardiovascular death (e.g., sepsis, cachexia) due to severe comorbidities. The observed lower mortality without a corresponding MACE reduction suggests GLP-1 RA use may be associated with lower mortality through non-atherosclerotic pathways, possibly involving anti-inflammatory effects 32 or enhanced resilience 33. However, this association was most pronounced early after initiation and attenuated over time, a pattern that may reflect treatment channeling toward clinically stable patients. Accordingly, we interpret these temporal findings cautiously as observational associations rather than definitive causal effects. The temporal attenuation of the mortality association in landmark analyses warrants cautious interpretation. While such a pattern might reflect a diminishing treatment effect over time, it could also arise from survivor-related or informative censoring bias, treatment channeling toward more clinically stable patients at initiation, or reduced statistical power due to smaller sample sizes at later time points.

### 4.3 Association with Aspiration Pneumonia Risk

We observed a lower incidence of aspiration pneumonia among GLP-1 RA initiators compared with DPP-4i initiators. Because aspiration pneumonia was identified using diagnosis codes in a frail population, outcome misclassification and differential ascertainment (e.g., variation in diagnostic workup, coding practices, and care intensity) cannot be excluded. In addition, the available data do not allow reliable classification of whether aspiration events were peri-procedural/anesthesia-related versus feeding-related. Therefore, mechanistic interpretations should be considered speculative. Recent peri-procedural studies reported no statistically significant increase in aspiration events associated with GLP-1 RA use; however, these studies examined associations in populations where full stomach precautions were commonly applied 34-37. The same caveat applies to our findings: because our database lacks granular data on peri-procedural management, the observed lower incidence of aspiration pneumonia among GLP-1 RA initiators may partly reflect clinical preventive measures rather than a pharmacological effect of GLP-1 RA 38.

However, our results provide observational evidence suggesting that delayed gastric emptying does not necessarily translate to an increased risk of clinical aspiration pneumonia in this population. This finding is consistent with the conclusions of several recent systematic reviews and meta-analyses, which, despite confirming that GLP-1 RA delay gastric emptying, did not demonstrate a statistically significant association between their use and the clinical incidence of aspiration pneumonia in patients undergoing elective surgery or endoscopic procedures. Our study extends this observation from the short-term perioperative setting to long-term outpatient follow-up in a high-risk population, further contributing this perspective to the existing body of observational evidence 37.

Several hypotheses could potentially explain this unexpected association, though these remain speculative. Pneumonia is fundamentally an inflammatory lung injury, and its risk and severity depend not only on the aspiration event itself but also on the host's immune response. GLP-1 RA possess systemic anti-inflammatory properties, capable of modulating the immune response, suppressing the production of pro-inflammatory cytokines (e.g., TNF-α and IL-6), and inhibiting the NF-κB inflammatory pathway 39. One hypothesis is that while GLP-1 RA may not reduce micro-aspiration events, their well-documented anti-inflammatory properties might theoretically reduce the likelihood of such events progressing to clinically significant pneumonia. However, this remains highly speculative, and the exact mechanisms underlying our observed association require further investigation through dedicated mechanistic studies. This observation suggests that the theoretical concerns regarding GLP-1 RA-related aspiration risk may not translate into clinical harm in this population, though this interpretation requires validation in future studies.

### 4.4 Interpretation of the Neutral MACE Outcome

Our neutral MACE findings contrast with meta-analyses showing 12-14% MACE reduction with GLP-1 RA versus placebo and superiority over DPP-4i 29, 31. Three factors explain this: First, using active comparator versus placebo reduces effect size. DPP-4i, though neutral in CVOTs 39, increase endogenous GLP-1 levels 40, 41 and outperform sulfonylureas. Second, high competing non-cardiovascular mortality (131 deaths vs. 136 MACE in DPP-4i group) limits MACE detection in frail populations. Third, insufficient power with 364 patients per group and < 2 years follow-up challenged detecting modest differences. The wide confidence interval (0.79-1.28) supports this.

### 4.5 Subgroup Analyses and Mechanistic Plausibility

The association with lower all-cause mortality was consistent across most pre-specified subgroups, supporting the consistency of the primary finding. Notably, this association appeared more pronounced in patients with a history of heart failure (aHR: 0.43, 95% CI: 0.27-0.69).

This finding has an important clinical context. Previously, CVOTs of DPP-4i raised concerns about a potential increased risk of hospitalization for heart failure (HHF), particularly for saxagliptin, as reported in meta-analyses of DPP-4i trials 42, although subsequent trials with other agents yielded neutral results. In contrast, meta-analyses of GLP-1 RA have shown a modest but significant reduction in HHF risk 43. Our observation of a notable association with reduced mortality in patients with a history of heart failure suggests that in a head-to-head comparison, the properties of GLP-1 RA on pathways relevant to heart failure (e.g., weight loss, blood pressure reduction, and potential direct myocardial effects) may be associated with more differential outcomes compared to the neutral effects of DPP-4i in this high-risk subgroup.

### 4.6 Exploration of MACE Outcomes in Younger Patients

Our exploratory analysis identified a potential signal for increased MACE risk among patients <65 years. This finding requires careful interpretation within the specific context of our post-gastrostomy population. While GLP-1 RA have established cardiovascular associations in the general adult population with type 2 diabetes 44, the interaction between age and underlying gastrointestinal pathology in determining cardiovascular risk remains poorly understood. The theoretical concerns about delayed gastric emptying and potential complications in patients with altered gastrointestinal anatomy 45, 46 may be particularly relevant in this subgroup. However, given the exploratory nature of this analysis, this finding should be considered hypothesis-generating, revealing an important knowledge gap that requires confirmation through targeted prospective research.

### 4.7 Strengths

This study addresses an important clinical question in a population systematically excluded from randomized controlled trials, using a target trial emulation framework with an active comparator design that reflects real-world treatment decisions. The extensive propensity score matching on multiple covariates represents a comprehensive attempt to address measured confounding. Several methodological approaches enhance the robustness of the analysis, including landmark analyses to assess temporal patterns, E-value calculations to quantify sensitivity to unmeasured confounding, and multiple sensitivity analyses including per-protocol approaches. The validation strategy using positive and negative controls (e.g., replicating known statin mortality benefits while showing no association with vitamin C) suggests the basic validity of the analytical platform and reduces concerns about systematic database bias.

### 4.8 Limitations

Critical limitations include: First, despite extensive PSM, unmeasured confounding remains likely. Unavailable variables include gastrostomy indication, functional status, nutritional parameters, frailty scores, and disease severity (e.g., cancer staging). These factors are fundamental determinants of outcomes in post-gastrostomy patients and may be strongly associated with treatment selection. Specifically, the database lacks structured information on the indication for gastrostomy, neurologic disease severity, functional/frailty status, swallowing function assessments, cancer staging, and goals of care, all of which may strongly influence both treatment selection and outcomes. Clinicians may preferentially prescribe GLP-1 RA to more clinically stable patients, introducing confounding by indication that propensity score matching cannot fully address. Although the E-value (2.17 for the point estimate; 3.11 for the confidence interval limit) suggests that a moderately strong unmeasured confounder would be needed to nullify the observed association, this threshold could plausibly be exceeded by unmeasured factors in this complex population.

Second, our study design limitations significantly impact the interpretation of secondary endpoints. With 364 patients per group and median follow-up of less than 2 years, the study was designed primarily for the mortality endpoint. The wide confidence intervals for MACE outcomes (0.79-1.28 for overall analysis) reflect substantial uncertainty and limit our ability to draw definitive conclusions about cardiovascular safety, particularly given the active comparator design where smaller effect sizes would be expected.

Third, our subgroup analyses were exploratory and should be considered hypothesis-generating rather than confirmatory. The potential MACE signal in patients < 65 years (aHR: 1.75, 95% CI: 1.05-2.92) occurred in the context of multiple subgroup comparisons, increasing the likelihood that this represents a chance finding rather than a true effect.

Fourth, the high competing mortality risk in our population (131 deaths vs. 136 MACE events in the DPP-4i group) may mask true associations with non-fatal outcomes. Although composite endpoints were used to partially address this bias, traditional competing-risk analyses (e.g., Fine-Gray subdistribution hazard models) were not feasible due to platform constraints described in the Methods section.

Fifth, outcome misclassification is an inherent limitation of database studies. Our reliance on ICD-10 coding for complex outcomes such as aspiration pneumonia and MACE lacks clinical validation or adjudication. The counterintuitive finding of lower pneumonia risk with GLP-1 RA use may partly reflect coding inconsistencies rather than true clinical benefit. For aspiration pneumonia in particular, reliance on diagnostic codes and the inability to adjudicate peri-procedural versus feeding-related timing may contribute to misclassification and differential ascertainment.

Finally, the generalizability of findings from this highly selected population to broader clinical practice remains uncertain. Post-gastrostomy patients represent a unique subset of the diabetes population, and our findings may not apply to other clinical contexts.

## 5. Conclusion

This target trial emulation found that GLP-1 RA initiation, compared with DPP-4i, was associated with lower all-cause mortality and aspiration pneumonia risk. However, given the limited sample size, these results warrant confirmation in prospective studies.

## Supplementary Material

Supplementary figure and tables.

## Figures and Tables

**Table 1:** Baseline Characteristics of Patients Treated with GLP-1 RA or DPP-4i Before and After PSM

	Before PSM			After PSM		
Characteristics	GLP-1 RA (N= 377)	DPP-4i (N= 1,171)	SMD	GLP-1 RA (N= 364)	DPP-4i (N= 364)	SMD
**Demographics**						
Age at Index	62.9±11.9	67.2±11.5	0.3699	63.2±11.7	63.5±11.7	0.0221
**Sex**						
Male	217(57.6%)	679(58.0%)	0.0086	212(58.2%)	224(61.5%)	0.0673
Female	155(41.1%)	474(40.5%)	0.0129	147(40.4%)	136(37.4%)	0.0620
**Race**						
White	242(64.2%)	594(50.7%)	0.2749	235(64.6%)	232(63.7%)	0.0172
Black	63(16.7%)	284(24.3%)	0.1877	63(17.3%)	58(15.9%)	0.0369
Asian	13(3.4%)	119(10.2%)	0.2690	13(3.6%)	16(4.4%)	0.0422
Unknown Race	36(9.5%)	89(7.6%)	0.0696	31(8.5%)	32(8.8%)	0.0098
Other Race	16(4.2%)	48(4.1%)	0.0073	15(4.1%)	19(5.2%)	0.0521
**Lifestyles**						
Nicotine dependence	84(22.3%)	225(19.2%)	0.0757	80(22.0%)	82(22.5%)	0.0132
Alcohol abuse	21(5.6%)	67(5.7%)	0.0066	20(5.5%)	22(6.0%)	0.0236
BMI	30.5±7.8	27.3±7.5	0.4077	30.5±7.7	28.3±7.4	0.0342
**Social economic status**						
Socioeconomic Hazards	30(8.0%)	74(6.3%)	0.0637	25(6.9%)	27(7.4%)	0.0213
**Comorbidities**						
Hypertension	339(89.9%)	1068(91.2%)	0.0439	329(90.4%)	331(90.9%)	0.0189
Hyperlipidemia	270(71.6%)	809(69.1%)	0.0555	259(71.2%)	265(72.8%)	0.0367
Cerebrovascular diseases	184(48.8%)	632(54.0%)	0.1035	178(48.9%)	189(51.9%)	0.0605
Ischemic heart diseases	175(46.4%)	544(46.5%)	0.0007	171(47.0%)	184(50.5%)	0.0715
GERD	173(45.9%)	474(40.5%)	0.1094	166(45.6%)	174(47.8%)	0.0441
Cancer	159(42.2%)	497(42.4%)	0.0054	156(42.9%)	149(40.9%)	0.0390
Malnutrition	145(38.5%)	525(44.8%)	0.1295	144(39.6%)	144(39.6%)	0.0000
CKD	134(35.5%)	490(41.8%)	0.1296	130(35.7%)	134(36.8%)	0.0229
Pneumonitis	103(27.3%)	353(30.1%)	0.0624	97(26.6%)	102(28.0%)	0.0308
COPD	76(20.2%)	236(20.2%)	0.0001	75(20.6%)	79(21.7%)	0.0269
Inflammatory arthritis	56(14.9%)	179(15.3%)	0.0121	53(14.6%)	55(15.1%)	0.0155
Osteoporosis	49(13.0%)	151(12.9%)	0.0030	46(12.6%)	47(12.9%)	0.0082
IBD	41(10.9%)	139(11.9%)	0.0313	39(10.7%)	32(8.8%)	0.0649
Intracranial injury	39(10.3%)	123(10.5%)	0.0052	34(9.3%)	41(11.3%)	0.0633
Dementia	20(5.3%)	160(13.7%)	0.2882	20(5.5%)	24(6.6%)	0.0461
Liver cirrhosis	16(4.2%)	50(4.3%)	0.0013	16(4.4%)	15(4.1%)	0.0136
Spine fracture	13(3.4%)	54(4.6%)	0.0592	13(3.6%)	17(4.7%)	0.0553
Parkinson's disease	10(2.7%)	43(3.7%)	0.0583	10(2.7%)	10(2.7%)	0.0000
SLE	10(2.7%)	10(0.9%)	0.1374	10(2.7%)	10(2.7%)	0.0000
**Medications**						
Analgesics	363(96.3%)	1133(96.8%)	0.0256	350(96.2%)	347(95.3%)	0.0408
Antithrombotic agents	362(96.0%)	1107(94.5%)	0.0701	349(95.9%)	349(95.9%)	0.0000
Insulin	354(93.9%)	1085(92.7%)	0.0497	342(94.0%)	342(94.0%)	0.0000
Statin	308(81.7%)	900(76.9%)	0.1196	299(82.1%)	296(81.3%)	0.0213
Beta blocking agents	308(81.7%)	958(81.8%)	0.0029	298(81.9%)	304(83.5%)	0.0436
Proton pump inhibitors	296(78.5%)	856(73.1%)	0.1267	286(78.6%)	289(79.4%)	0.0202
Diuretics	294(78.0%)	840(71.7%)	0.1445	282(77.5%)	284(78.0%)	0.0132
Corticosteroids	281(74.5%)	769(65.7%)	0.1946	271(74.5%)	264(72.5%)	0.0436
Benzodiazepine derivatives	281(74.5%)	837(71.5%)	0.0689	270(74.2%)	272(74.7%)	0.0126
Dihydropyridine derivatives	218(57.8%)	657(56.1%)	0.0347	211(58.0%)	210(57.7%)	0.0056
Metformin	220(58.4%)	532(45.4%)	0.2609	208(57.1%)	209(57.4%)	0.0056
ACEI	167(44.3%)	515(44.0%)	0.0064	165(45.3%)	158(43.4%)	0.0387
metoclopramide	132(35.0%)	366(31.3%)	0.0799	129(35.4%)	130(35.7%)	0.0057
**Laboratory Tests**						
Platelets	285.0±110.5	271.8±117.3	0.1156	282.5±109.6	282.4±121.2	0.0408
Albumin	3.3±0.7	3.1±0.7	0.2346	3.2±0.7	3.1±0.7	0.0000
eGFR	80.8±29.8	77.8±31.8	0.0968	79.8±29.8	82.4±32.6	0.0289
HbA1c	7.8±2.0	7.3±1.9	0.2198	7.8±2.0	7.3±2.0	0.0128
CRP	51.7±68.6	56.9±70.9	0.0746	53.8±69.8	48.9±59.5	0.0449

**Table 2:** Overall and Landmark Hazard Ratios for MACE and All-Cause Mortality in the Matched Cohort

	Post-matching Analysis					Landmark analyses ^c^		
Outcomes	GLP-1 RA (n=364)	DPP-4i (n=364)	Overall HR (95%CI)^ a^	Overall E-value^ b^		>30 days HR (95% CI)	>60 days HR (95% CI)	>180 days HR (95% CI)
				Point value	95% CI Limit			
**All-cause Mortality**	91	131	0.71 (0.54 -0.93)	2.17	3.11	0.63 (0.44-0.89)	0.79 (0.52-1.21)	0.99 (0.62-1.56)
**MACE**	135	136	1.01(0.79 -1.28)	1.11	1.00	0.96 (0.68-1.36)	0.70 (0.45-1.08)	1.13 (0.67-1.91)
**Aspiration Pneumonia**	47	71	0.64(0.44 -0.93)	2.50	3.97	0.64 (0.40-1.02)	0.81 (0.48-1.37)	0.77 (0.45-1.32)

^a^ Propensity score matching was performed on all listed characteristics. ^b^ The E-value represents the minimum strength of association that an unmeasured confounder would need to have with both the exposure and the outcome to fully explain the observed hazard ratio. In this analysis, the E-value was 2.17 for all-cause mortality, 1.11 for major adverse cardiovascular events (MACE), and 2.50 for aspiration pneumonia, suggesting that the findings are relatively robust to potential unmeasured confounding. ^c^ A landmark approach was used to analyze risk at different time points. The >30 days analysis included only patients who were alive and event-free at day 31 to assess their subsequent risk. This was similarly applied to the >60 days and >180 days analyses, which respectively included event-free survivors at day 61 and day 181. * The mean (±SD) follow-up duration was 3.53 (±3.63) years in the GLP-1 RA group and 2.85 (±3.06) years in the DPP-4i group. The median follow-up times were 1.93 and 1.73 years, respectively. **Abbreviations:** CI, confidence interval; DPP-4i, dipeptidyl peptidase-4 inhibitors; GLP-1 RA, glucagon-like peptide-1 receptor agonists; HR, hazard ratio; MACE, major adverse cardiovascular events.

## Data Availability

The data source for this study was the TriNetX global collaborative health research network. This is a federated database of de-identified electronic health records that provides longitudinal clinical data, including patient demographics, diagnoses, procedures, laboratory results, and prescribed medications.
