# Supplementary material for: Association of Glucagon-like Peptide-1 Receptor Agonists with Mortality and Aspiration Pneumonia in Patients with Type 2 Diabetes After Gastrostomy: A Target Trial Emulation Study
Source: Int J Med Sci. 2026 Mar 17;23(4):1444–55. doi: 10.7150/ijms.128956 (PMC13048897; doi:10.7150/ijms.128956)
Supplement: Supplementary file 1 — Supplementary figure and tables. [file ijmsv23p1444s1.pdf]

Supplementary

Supplementary eTable 1. Definitions and Codes for Exposures, Outcomes, and Covariates

Variable Category	Variable Name	Definition / Codes Used
Index / Eligibility	PEG procedure	ICD-10-PCS: 0DH63UZ, 0DH64UZ (Percutaneous / Endoscopic Approach)
	Exposure A (GLP-1)	ATC: A10BJ (GLP-1 analogues)
	Exposure B (DPP-4i)	ATC: A10BH (DPP-4 inhibitors)
	Age ≥18 at event	TNX:Visit (≥18 years old at event)
Demographics	Age at Index	Continuous variable (years)
	Sex	Female (F), Male (M)
	Race	White (2106-3), Black (2054-5), Asian (2028-9), Other (2131-1), Unknown (UNK)

Comorbidities	Hypertensive diseases	ICD-10-CM: I10–I1A
	Ischemic heart diseases	ICD-10-CM: I20–I25
	Cerebrovascular diseases	ICD-10-CM: I60–I69
	Chronic kidney disease (CKD)	ICD-10-CM: N18
	Malnutrition	ICD-10-CM: E40–E46
	COPD	ICD-10-CM: J44
	GERD	ICD-10-CM: K21
	Neoplasms	ICD-10-CM: C00–D49
	Inflammatory polyarthropathies	ICD-10-CM: M05–M14

Bone density/structure disorders	ICD-10-CM: M80–M85
Dementia (unspecified)	ICD-10-CM: F03
Parkinson’s disease	ICD-10-CM: G20
Systemic lupus erythematosus (SLE)	ICD-10-CM: M32
Intracranial injury	ICD-10-CM: S06
Fracture of lumbar spine & pelvis	ICD-10-CM: S32
Nicotine dependence	ICD-10-CM: F17
Personal history of nicotine dependence	ICD-10-CM: Z87.891

	Alcohol abuse	ICD-10-CM: F10.1
	Socioeconomic hazards	ICD-10-CM: Z55–Z65
Medications	Analgesics	ATC: CN100
	Antithrombotic agents	ATC: B01A
	Proton pump inhibitors (PPIs)	ATC: A02BC
	Corticosteroids	ATC: R01AD
	Benzodiazepine derivatives	ATC: N05CD
	Metoclopramide	RxNorm: 6915
	Metformin	RxNorm: 6809
	Insulin	HS501

	ACE inhibitors, plain	ATC: C09A
	Beta-blocking agents	ATC: C07
	Dihydropyridine derivatives	ATC: C08CA
	Diuretics	ATC: C03
	Statins (HMG-CoA reductase inhibitors)	ATC: C10AA
Laboratory Tests	BMI	TNX: 9083 (kg/m^2)
	Platelets	TNX: 9020 ($10^3/\mu L$)
	Albumin	TNX: 9045 (g/dL)
	C-reactive protein (CRP)	TNX: 9063 (mg/L)
	Hemoglobin A1c	TNX: 9037 (%)

	eGFR (CKD-EPI 2021)	LOINC: 98979-8 (mL/min/1.73m^2)
Outcomes	All-cause mortality	Deceased (database record)
	Aspiration pneumonia	ICD-10-CM: J69
	MACE	ICD-10-CM: I21, I46, I61, I63
	Acute pancreatitis	ICD-10-CM: K85
	Biliary-related diseases	ICD-10-CM: K80–K83
	Diseases of appendix	ICD-10-CM: K35–K38
	GI adverse events	ICD-10-CM: K31.84, K56, R11, K91.3
	Urinary system disorders	ICD-10-CM: N39

Supplementary eTable 2. Risk of All-Cause Mortality, MACE, and Pneumonia with Different Covariate Adjustment Models

Outcomes	Model 1[a]	Model 2[b]	Model 3[c]	Model 4[d]	Model 5[e]
All-cause mortality	0.63 (0.50–0.79)	0.76 (0.58–1.01)	0.66 (0.50–0.86)	0.75 (0.56–0.99)	0.71 (0.54–0.93)
MACE	0.93 (0.77–1.13)	1.19 (0.93–1.53)	1.06 (0.83–1.35)	1.12 (0.87–1.43)	1.01 (0.79–1.28)
Aspiration Pneumonia	0.57 (0.41–0.77)	0.64 (0.44–0.92)	0.66 (0.46–0.96)	0.69 (0.48–1.00)	0.64 (0.44–0.93)

CI: Confidence interval.

[a] Crude, before matching.

[b] Propensity score matching was performed on age at index, sex, race.

[c] Propensity score matching was performed on age at index, sex, race, social economic status, lifestyles, medications.

[d] Propensity score matching was performed on age at index, sex, race, social economic status, lifestyles, medications, comorbidities.

[e] Propensity score matching was performed on all listed characteristics.

Supplementary eTable 3. Comparison of intention-to-treat (ITT) and per-protocol (PP) Analyses for Clinical Outcomes.

		GLP-1	DPP-4	
Outcome	Analysis Type	N (Events)	N (Events)	aHR (95% CI)
All-Cause Mortality	ITT	364 (91)	364 (131)	0.71 (0.54–0.93)
	PP	284(72)	284(115)	0.64 (0.48–0.87)
MACE	ITT	364 (135)	364 (136)	1.01 (0.79–1.28)
	PP	284(102)	284(111)	0.92 (0.70–1.21)
Aspiration Pneumonia	ITT	364 (47)	364 (71)	0.64 (0.44–0.93)
	PP	284(36)	284(68)	0.52 (0.35–0.78)

Data are presented as adjusted hazard ratios (aHRs) with 95% confidence intervals (CIs). The ITT analysis included all patients after propensity score matching. The per-protocol analysis excluded patients who switched from their initial treatment group to the comparator's drug class

during follow-up. N (Events) indicates the total number of patients in the group for the analysis and the number of patients who experienced the outcome.

Abbreviations: aHR, adjusted hazard ratio; CI, confidence interval; DPP-4, dipeptidyl peptidase-4; GLP-1, glucagon-like peptide-1; ITT, intention-to-treat; MACE, major adverse cardiovascular events; PP, per-protocol.

Variables	aHR (95% CI)
**Primary outcome**	
All−cause mortality	0.71 (0.54 − 0.93)
**Secondary outcome**	
MACE	1.01 (0.79 − 1.28)
Aspiration Pneumonia	0.64 (0.44 − 0.93)
**Composite Outcome Analyses**	
MACE or All−cause mortality	0.93 (0.76 − 1.14)
Pneumonia or All−cause mortality	0.74 (0.58 − 0.95)
**Positive outcome control**	
Biliary−related diseases	0.96 (0.62 − 1.46)
Gastrointestinal adverse events	0.81 (0.62 − 1.06)
**Negative outcome control**	
Appendicitis	2.05 (0.19 − 22.60)
Urinary tract infection	0.76 (0.58 − 1.01)
Acute Pancreatitis	0.50 (0.17 − 1.47)
**Positive exposure control (Statin)**	
All−cause mortality	0.75 (0.69 − 0.82)
MACE	1.05 (0.85 − 1.30)
Aspiration Pneumonia	1.08 (0.96 − 1.21)
**Negative exposure control (Vitamin C)**	
All−cause mortality	0.93 (0.86 − 1.00)
MACE	1.01 (0.94 − 1.10)
Aspiration Pneumonia	0.94 (0.84 − 1.04)

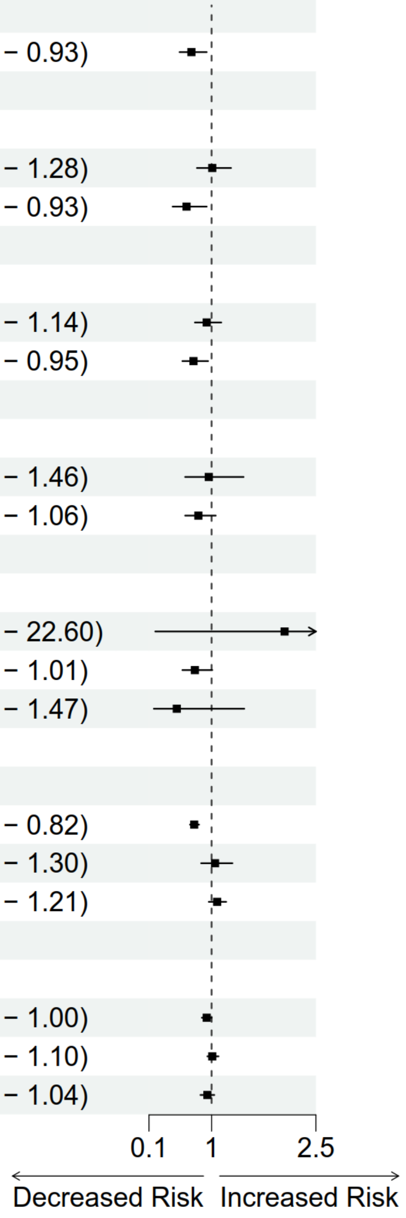

Supplementary eFigure 1: Differential Clinical Outcomes Between GLP-1 and DPP-4 Inhibitor Users: A Validation Analysis Including Both Outcome and Exposure Controls.
